# Giant pedunculated hepatoblastoma mimicking neuroblastoma in a 4-month-old infant: a case report

**DOI:** 10.1186/s12887-023-03954-x

**Published:** 2023-03-30

**Authors:** Elham Zarei, Mehdi Vafadar, Mohammad Saeid Khonji, Amir Sajjad Mounesi Sohi

**Affiliations:** 1 School of Medicine, Ali Asghar Children Hospital, Iran University of Medical Sciences, Tehran, Iran; 2 Bone and Joint Reconstruction Research Center, Iran University of Medical Sciences, Tehran, Iran; 3 Department of Radiology, School of Medicine, Iran University of Medical Sciences, Tehran, Iran

**Keywords:** Hepatoblastoma, Pedunculated, Diagnosis, Imaging

## Abstract

**Background:**

Hepatoblastoma is the most common primary malignancy of hepatic origin in children, with an estimated incidence of 0.5–1.5 per million children. Hepatoblastoma classically has an intraparenchymal location, and pedunculated hepatoblastoma is a relatively rare entity. Accurate diagnosis can be challenging due to its extrahepatic location and possibly its thin peduncle, which is not easily identified in imaging.

**Case presentation:**

Here, we report a case of asymptomatic giant palpable hepatoblastoma in the LUQ of a four-month-old male infant, initially suspected of neuroblastoma based on abdominal ultrasound findings. The final diagnosis of giant pedunculated hepatoblastoma was made based on the abdominal CT scan and the diagnosis was confirmed by percutaneous biopsy. Due to the size of the tumor, complete removal of the tumor was not initially possible. Therefore, the patient was treated with several courses of chemotherapy. The tumor was shrunk and then completely removed. The patient was treated, and no complications were found in the 6-month follow-up.

**Conclusion:**

Pedunculated hepatoblastoma is rare but should be considered as a possibility in the case of a perihepatic mass in a pediatric patient that can be confused with other upper abdominal masses such as an adrenal mass. Therefore, in such cases, we must look for the vascular pedicle in the imaging and keep the AFP check in mind.

## Introduction

Hepatoblastoma is the most common primary malignancy of liver origin in children, with an estimated incidence of 0.5–1.5 per million children from birth to 14 years of age [1]. It occurs more in males than females with a M:F = 1.6:1.0 [2]. The congenital form of hepatoblastoma, which accounts for less than 10% of pediatric hepatoblastomas, is found in utero or within the first 28 days after birth [3, 4]. In infants, hepatoblastoma usually presents with acute abdominal distension and palpable abdominal mass, and respiratory distress [5, 6].

Pediatric hepatoblastoma is diagnosed using laboratory markers and imaging studies, but a definitive diagnosis is based on pathological examination [1]. A common tumor marker for hepatoblastoma screening and diagnosis is alpha-fetoprotein (AFP). It increases in approximately 90% of cases. However, AFP is not sensitive or specific to hepatoblastoma and is also elevated in other liver malignancies [1]. Beta-Human Chorionic Gonadotropin (β-HCG) is increased in about 20% of patients [2]. Ultrasound and CT scans are usually performed on children suspected of having abdominal masses. These imaging studies are critical to narrow down the differential diagnosis.

Pedunculated hepatoblastoma is a relatively rare subtype of hepatoblastoma that is characterized by a tumor mass attached to the liver by a pedicle or stalk and to the best of our knowledge, few cases have been reported in the literature [7, 8].

The diagnosis of pedunculated hepatoblastoma can be challenging as there is difficulty in determining the origin of the mass as the thin peduncle/stalk may be unrecognizable on imaging.

Here, we report a four-month-old male infant with a large, asymptomatic, palpable abdominal mass, initially thought to be neuroblastoma, but later diagnosed as a pedunculated hepatoblastoma.

## Case presentation

A four-month-old male infant was admitted for a palpable mass in the left upper quadrant (LUQ). The mass was first detected by his mother a week ago. The patient did not have any specific signs and symptoms and did not have failure to thrive or developmental delay. The mother’s pregnancy and delivery were uneventful. His birth weight was 2800 g. The CBC, and liver functions test, including liver enzymes, prothrombin time and partial thromboplastin time, bilirubin, blood urea, and creatinine were normal.

Abdominal ultrasound showed a large, slightly heterogeneous solid mass with clear borders containing scattered calcifications of approximate dimensions 70 × 55 × 40 mm in the LUQ, which was in front of the left kidney and inferior to the spleen and pancreas without a definite claw sign with any adjacent solid organs. Considering the infant’s age, the above findings led to the clinical suspicion of neuroblastoma. To rule in or rule out the diagnosis of neuroblastoma, bone marrow aspiration and 24-hour urine collection (VMA and HVA) were performed, which were negative.

**Fig. 1:**
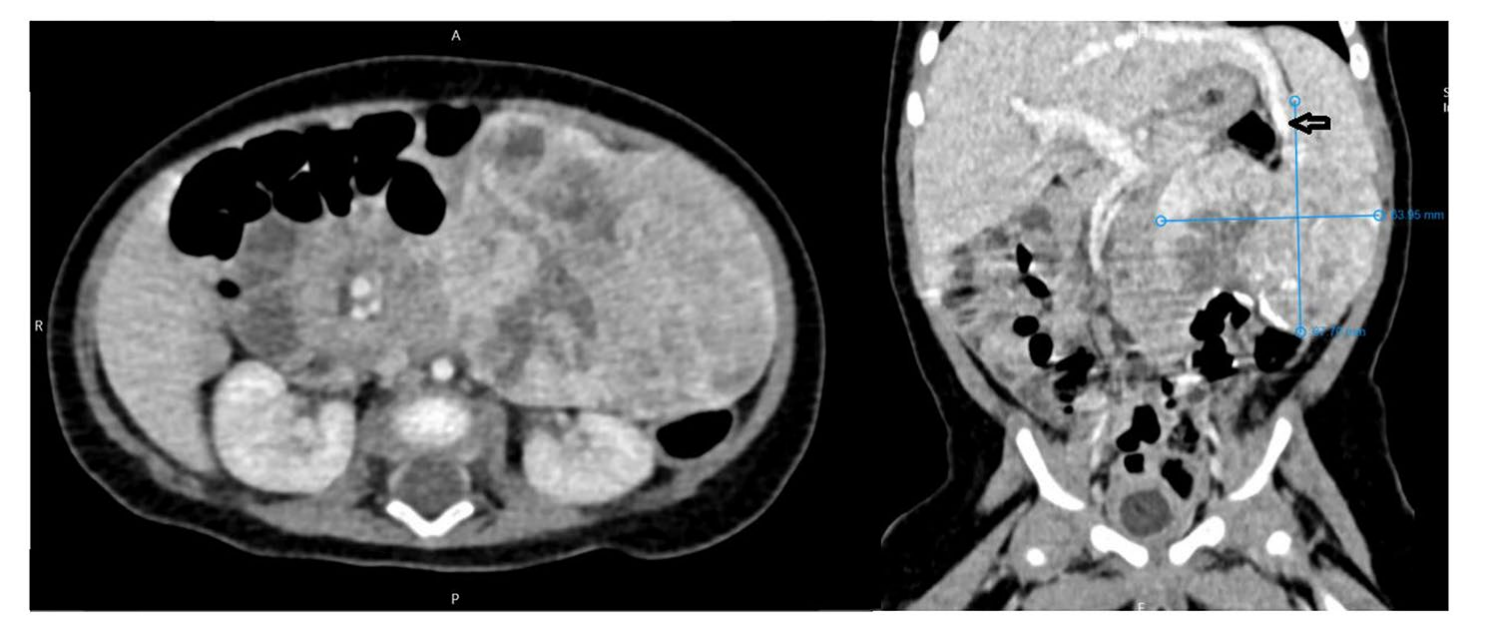
A well-defined slightly heterogeneous enhancing solid mass containing scattered calcifications of approximate dimensions 70x55x40 mm in the LUQ, which was in front of the left kidney and inferior to the spleen and pancreas without a definite claw sign with any adjacent solid organs was found. The arrow shows the tumor feeding vessels from segment III of the left liver lobe passing into the upper part of the above mass without peripheral tissue.

In the next step, a CT scan of the abdomen and pelvis with intravenous contrast was performed which confirmed the ultrasound findings. (Fig. 1)

On CT scan, a few vascular channels were identified from segment III of the left liver lobe passing into the upper part of the above mass without peripheral tissue.

**Fig. 2:**
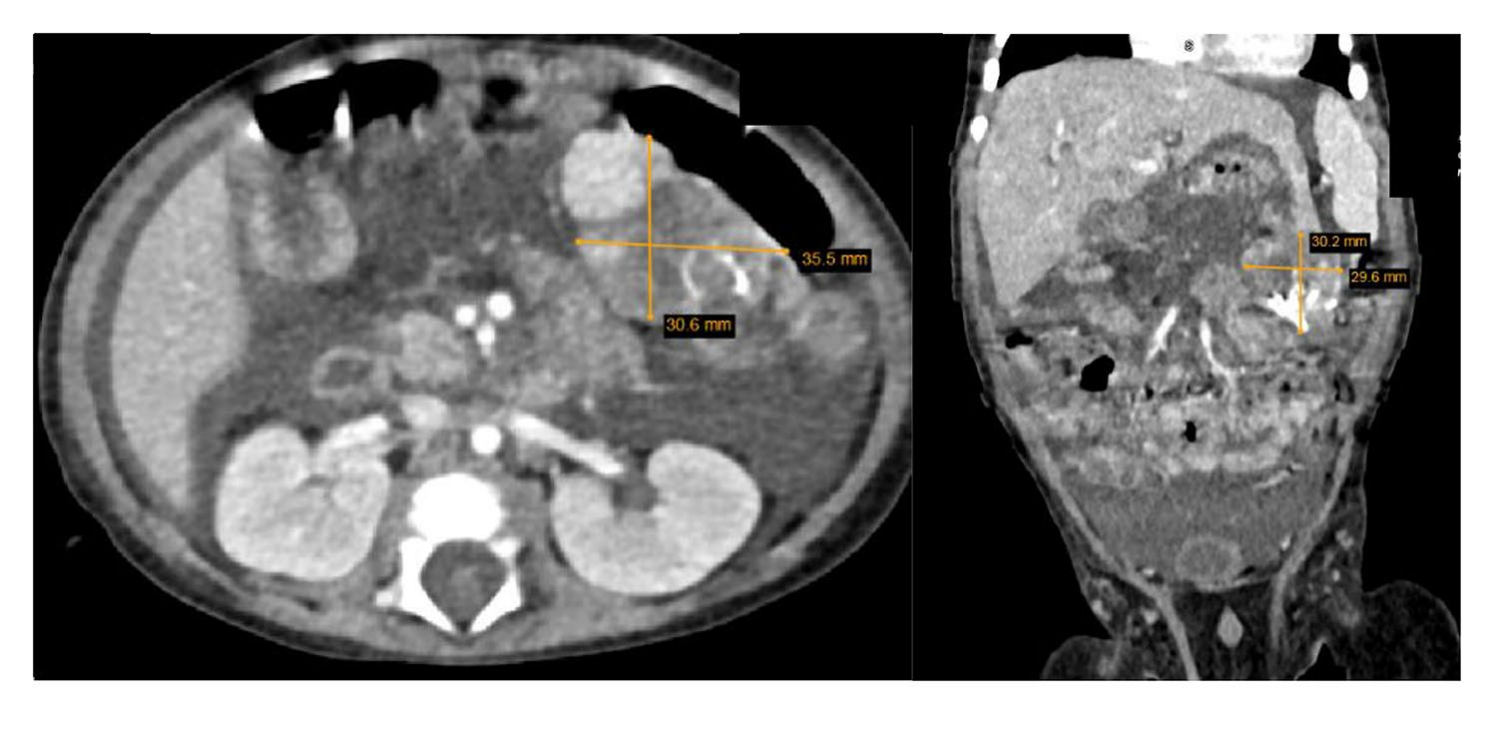
Epithelial type hepatoblastoma with post-chemotherapy changes (calcification, fibrosis and hemosiderin laden macrophages).

According to the negative results for neuroblastoma and considering the entry of nutrient vessels into the mass from the left hepatic vein and the coarse calcification areas and the pattern of enhancement, pedunculated hepatoblastoma was suggested and AFP was measured which was more than 100,000 ng/ml (normal 5-109). β-HCG was within the normal range. the patient subsequently underwent a core needle biopsy. The pathology report revealed fetal hepatocytes with enlarged nuclei, and abundant cytoplasm arranged in a light-dark pattern due to admixture of glycogenous and eosinophilic hepatocytes with rare mitosis, consistent with the diagnosis of epithelioid hepatoblastoma (Fig. 2). According to the PRETEXT classification, the tumor was classified as PRETEXT 1.

**Fig. 3:**
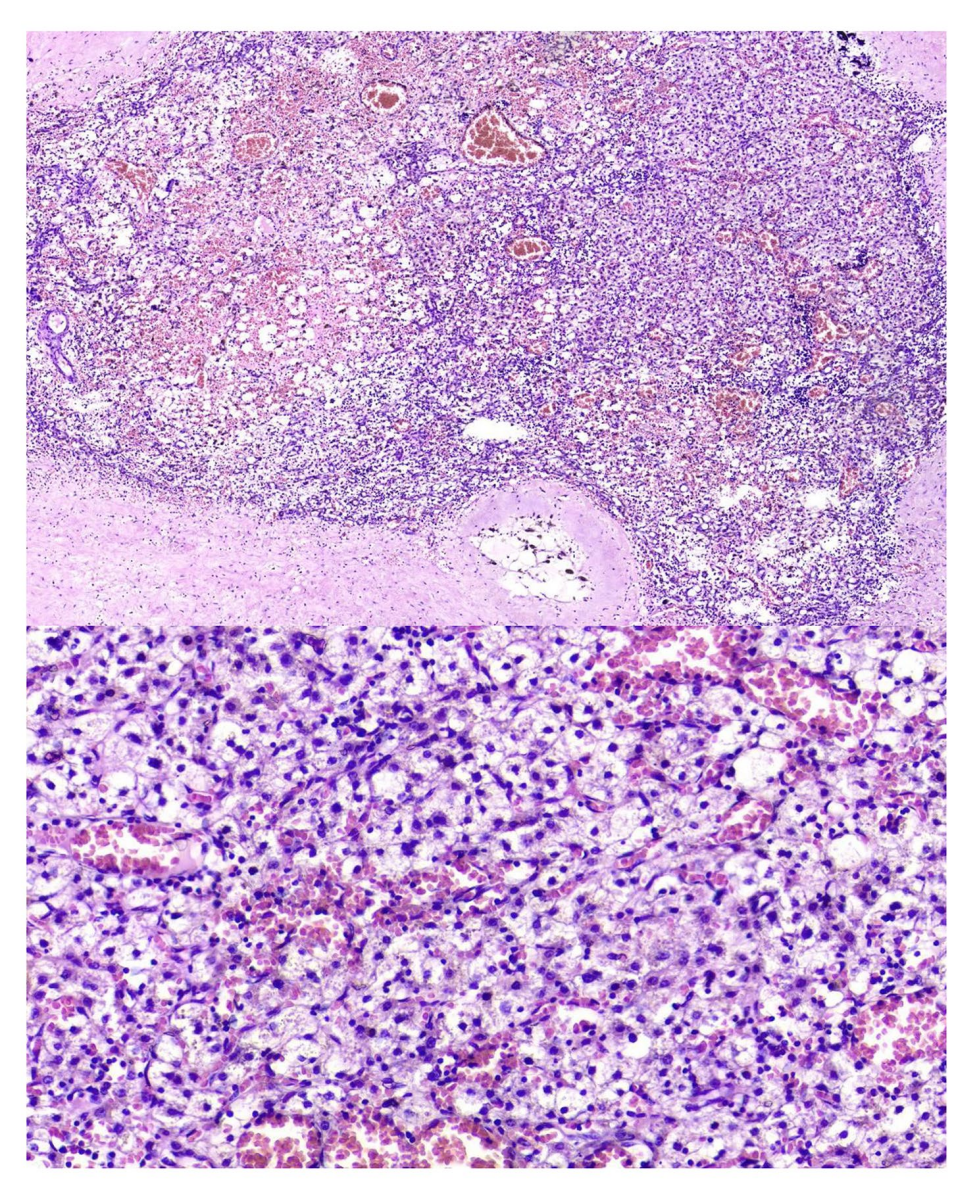
CT imaging after preoperative chemotherapy showing a significant reduction in the size of the tumor.

Due to the size of the tumor, complete removal of the tumor was not feasible at first. Therefore, the patient was treated with several courses of chemotherapy (Fig. 3). The preoperative chemotherapy regimen, which was given every 3 weeks, consisted of doxorubicin (25 mg/m2 × 3 days continuous infusion by central line) and cisplatin (20 mg/m2 × 5 days continuous infusion, days 0–4. After shrinking the tumor, the patient underwent surgery and the pedunculated mass with dimensions of approximately 20*40*40 mm, which had no adhesion anywhere, was resected along with the III segment of the left lobe of the liver. After surgery, the patient underwent 4 additional cycles of chemotherapy. He was symptom-free without complications during the 6-month follow-up.

## Discussion

Primary liver tumors account for 1–2% of all childhood tumors [9, 10]. Hepatoblastoma is a malignant liver tumor in young children that accounts for more than 90% of liver cancers in children under 5 years of age [11]. It mainly occurs as a single focus in the right lobe of the liver, but it can be multifocal and occur in all parts of the liver. The cause of hepatoblastoma, which usually occurs sporadically, is still unknown [2]. Combining complete resection of the primary tumor with chemotherapy is the best treatment for long-term cure [12]. If the hepatoblastoma is completely resectable, the prognosis is favorable, primarily because it responds well to adjuvant chemotherapy. If it cannot be completely removed the prognosis is much less favorable [13, 14]. About 12% of hepatoblastoma relapse after complete remission [2].

This report presents a case of a large pedunculated hepatoblastoma in an asymptomatic four-month-old infant who was initially treated with chemotherapy to make the tumor resectable. We found only two reports of pedunculated hepatoblastoma in the literature, one from the right lobe and the other from the hepatogastric ligament [7, 8].

These cases were 3 and 7-year-old children who presented with peduncle hepatoblastoma measuring 3 × 4 cm and 8.1 × 7.4 × 4.4 cm respectively [7, 8]. In Chen et al. study, pathology demonstrated hepatoblastoma in the hepatogastric ligament (subtype, mixed fetal and embryonal hepatoblastoma). They removed the tumor with surgical intervention. The patient underwent six cycles of postoperative chemotherapy. No sign of recurrence was found through 3 years after surgery. In Oshiro et al. study, the pathological assessment showed an epithelial type hepatoblastoma composed of different proportions of fetal and embryonal cells. However, they did not elaborate on treatment or prognosis of the case.

The case presented in this article is a four-month-old baby with a 70 × 55 × 40 mm mass, which is extremely large for an infant of this age.

Unlike pedunculated hepatoblastoma, several case reports of pedunculated hepatocellular carcinoma have been published, which may help us better understand pedunculated hepatoblastoma. Well-developed hepatoblastoma may mimic hepatocellular carcinoma.

The stalk transports nutrients from the liver and connects a tumor to the liver.

Without the protection of the liver parenchyma, a pedunculated liver tumor is vulnerable to bleeding, which rapidly leads to tumor spread [13, 14].

No bleeding or spread or relapse was found in our case.

## Conclusion

Pedunculated hepatoblastoma is rare but should be considered as a possibility in the case of a perihepatic mass in a pediatric patient that can be confused with other upper abdominal masses such as an adrenal mass. Therefore, in such cases, we must look for the vascular pedicle in the imaging and keep the AFP check in mind.

## Data Availability

Not applicable.

## Abbreviations

LUQ: left upper quadrant
β-HCG: Beta-Human Chorionic Gonadotropin
AFP: alpha-fetoprotein
